# Block copolymer conjugated Au-coated Fe_3_O_4_ nanoparticles as vectors for enhancing colloidal stability and cellular uptake

**DOI:** 10.1186/s12951-017-0290-5

**Published:** 2017-07-25

**Authors:** Junbo Li, Sheng Zou, Jiayu Gao, Ju Liang, Huiyun Zhou, Lijuan Liang, Wenlan Wu

**Affiliations:** 1 School of Chemical Engineering & Pharmaceutics, Henan University of Science & Technology, Luo Yang, 471023 China; 2 School of Medicine, Henan University of Science & Technology, Luo Yang, 471023 China

**Keywords:** Block copolymer, Heterogeneous nanoparticles, Magnetofection, Colloidal stability, Gene vector

## Abstract

**Background:**

Polymer surface-modified inorganic nanoparticles (NPs) provide a multifunctional platform for assisting gene delivery. Rational structure design for enhancing colloidal stability and cellular uptake is an important strategy in the development of safe and highly efficient gene vectors.

**Results:**

Heterogeneous Au-coated Fe_3_O_4_ (Fe_3_O_4_@Au) NPs capped by polyethylene glycol-*b*-poly1-(3-aminopropyl)-3-(2-methacryloyloxy propylimidazolium bromine) (PEG-*b*-PAMPImB-Fe_3_O_4_@Au) were prepared for DNA loading and magnetofection assays. The Au outer shell of the NPs is an effective platform for maintaining the superparamagnetism of Fe_3_O_4_ and for PEG-*b*-PAMPImB binding via Au–S covalent bonds. By forming an electrostatic complex with DNA at the inner PAMPImB shell, the magnetic nanoplexes offer steric protection from the outer corona PEG, thereby promoting high colloidal stability. Transfection efficiency assays in human esophageal cancer cells (EC109) show that the nanoplexes have high transfection efficiency at a short incubation time in the presence of an external magnetic field, due to increased cellular internalization via magnetic acceleration. Finally, after transfection with the magnetic nanoplexes EC109 cells acquire magnetic properties, thus allowing for selective separation of transfected cells.

**Conclusion:**

Precisely engineered architectures based on neutral-cationic block copolymer-conjugated heterogeneous NPs provide a valuable strategy for improving the applicability and efficacy of synthesized vectors.

**Electronic supplementary material:**

The online version of this article (doi:10.1186/s12951-017-0290-5) contains supplementary material, which is available to authorized users.

## Background

Gene therapy has emerged as a promising approach for delivering foreign nucleic acid into target cells for the treatment of cancer [1, 2]. Successful gene therapy requires safe and effective gene delivery systems to introduce genetic material into tissues or cells without causing harmful side effects [3, 4]. Although viral vectors remain the primary gene delivery system utilized in gene therapy, they present important limitations, including considerable immunogenicity, toxin production and limited size transgenic capacity [5]. Synthetic gene vectors offer unique advantages that resolve some of these technical hurdles, for instance, they have a modifiable structure, low immunogenic response and the capability to carry large inserts [6–8]. However, as most synthetic gene vectors have low colloidal stability and poor transfection efficiency, they have limited applications in vivo [9–11]. More recently, inorganic nanoparticles have provided attractive scaffolds to assist gene delivery due to their unique physical and chemical properties. Moreover, the controlled synthesis, assembly and modification of these particles have seen remarkable technical advances [12, 13]. Gold nanoparticles (Au NPs) show great potential for applications in gene delivery because they have controlled size, excellent biocompatibility, tailored surface chemistry and can be easily synthesized [14, 15]. It has been demonstrated that a variety of Au NPs with polycationic modifications can increase siRNA and plasmid DNA payload and thus enhance transfection efficiency, when compared to the surface materials alone [16, 17]. However, the exposed positive surface of these vectors causes undesired aggregation and extensive vector accumulation in the physiological media [18, 19], resulting in impaired intracellular uptake and hence unsuitability for in vivo applications [20]. To overcome these limitations, block copolymers have been introduced which enhance the colloidal stability of Au NP-based vectors by means of a novel three layer micelle-like structure [21]. For instance, poly(*N*-2-hydroxypropyl methacrylamide-*block*-*N*-[3-(dimethylamino)propyl] methacrylamide) [P(HPMA-*b*-DMAPMA)] stabilized Au NPs were developed by McCormick’s group for siRNA delivery [22]. The cationic PDMAPMA was used for binding siRNA, and the neutral PHPMA shell for protecting it against enzymatic degradation. Kataoka’s group fabricated a similar NP small vehicle (~50 nm) that showed significant colloidal stability in vivo and high accumulation of siRNA in a cancer model [23]. Moreover, our group studied a process of DNA loading and the influence of PEG-*b*-PAMPImB-capped Au NPs on colloid stability during delivery [24]. These vectors exhibited a mono-disperse state to translocate across the cell membrane and then partly entered the nucleus, thus inducing high and efficient gene expression. The neutral outer corona significantly promoted high colloidal stability, however, it also had a negative effect on cellular uptake due to the reduced interaction between vector and cell membrane, called “PEG dilemma” [25, 26].

Iron oxide magnetic nanoparticles (Fe_3_O_4_ NPs) have also become attractive nanomaterials with promising application prospects for gene delivery due to their recently discovered superparamagnetic behavior [27, 28]. With their polycationic surface and magnetic core, these vectors can carry nuclear acids to target cells within minutes when an external magnetic field is applied [29, 30]. Notably, cellular internalization is activated independently of the magnetic NPs’ surface properties, including neutral or negative charges [31, 32]. The efficiency of magnetofection is several hundredfolds higher than that of conventional transfections [33–35], however, Fe_3_O_4_ NPs present some disadvantages. For instance, while surface covalent bond modifications are difficult to achieve, surface oxidation and corrosion occur easily in physiological environments [36, 37]. Au-coated Fe_3_O_4_ heterogeneous NPs (Fe_3_O_4_@Au) have been widely explored for increasing Fe_3_O_4_ core stability and biocompatibility. These NPs provide a platform for easily achieving covalent modifications via Au–S chemistry, while maintaining their magnetic targeting function [38]. Moreover, Fe_3_O_4_@Au NPs may have applications in bioseparation [39], bioimaging [40], and photodynamic therapy of cancer [41].

**Scheme 1:**
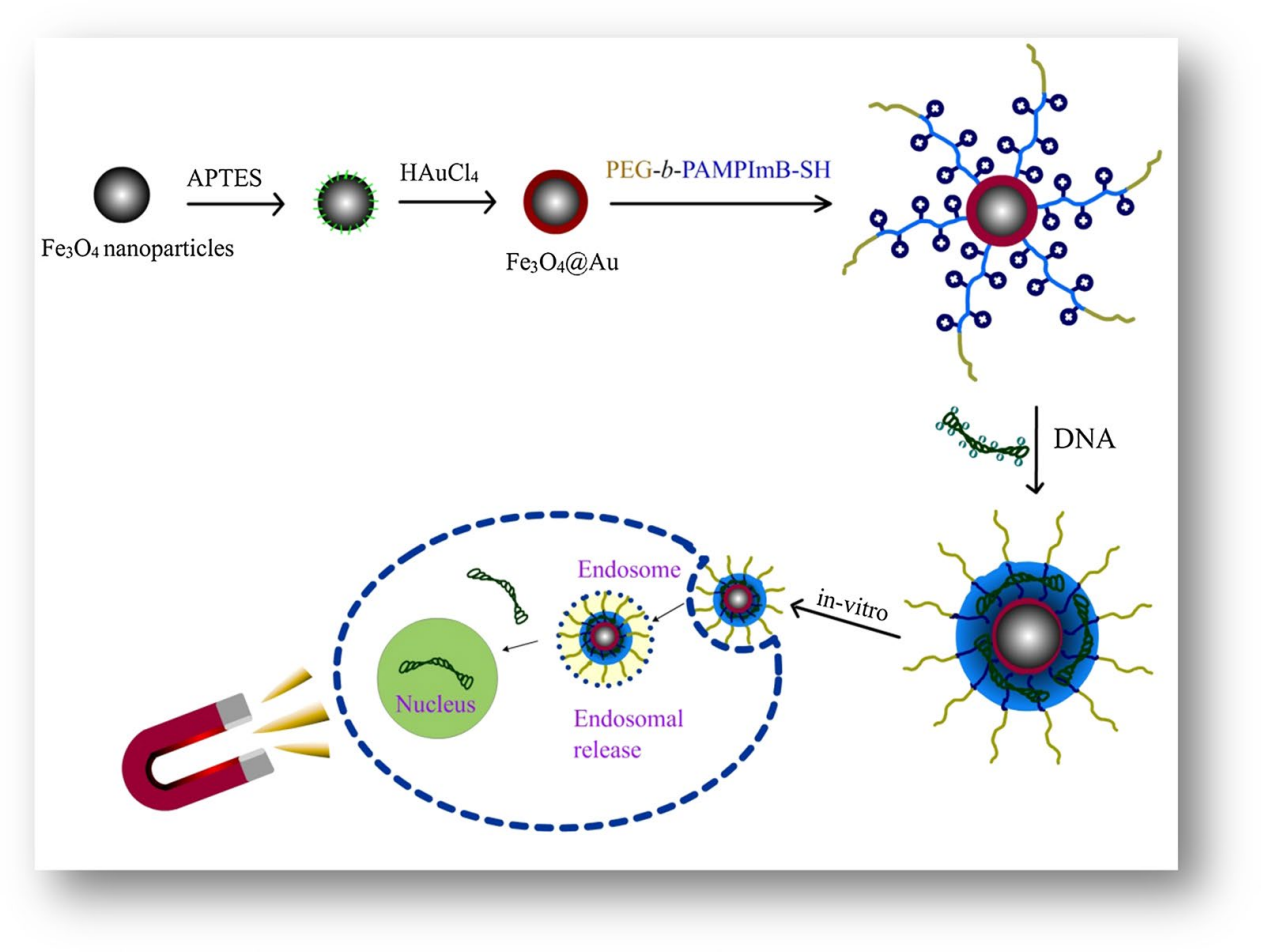
Schematic representation of the magnetic vector and magnetofection process

Here, heterogeneous Fe_3_O_4_@Au NPs conjugated to block copolymer PEG-*b*-PAMPImB were prepared for DNA delivery and magnetofection assays. The vector was constructed with four layers: (i) a Fe_3_O_4_ NP core, to increase cellular internalization through magnetic acceleration; (ii) an outer Au shell, to facilitate binding with block copolymer via Au–S bonds; (iii) an inner PAMPImB block, for condensing DNA; and (iv) a PEG corona, to increase colloidal stability. The synthesis of the magnetic vector and magnetofection process are illustrated in Scheme 1.

## Results and discussion

### Preparation and characterization of PEG-*b*-PAMPImB-Fe_3_O_4_@Au

We prepared heterogeneous Fe_3_O_4_@Au NPs by using a widely reported and efficient approach [42]. APTES functional Fe_3_O_4_ NPs were used as heterogeneous nucleation sites and AuCl_4_^−^ was then directly reduced on the interface of those Fe_3_O_4_ NPs. Next, we converted the dithioester of PEG-*b*-PAMPImB [previously synthesized by reversible addition fragmentation chain transfer (RAFT) polymerization] into a thiol end-group with hydrazine, and obtained PEG-*b*-PAMPImB-Fe_3_O_4_@Au NPs via a strong binding affinity between gold and thiol ending.

**Fig. 1:**
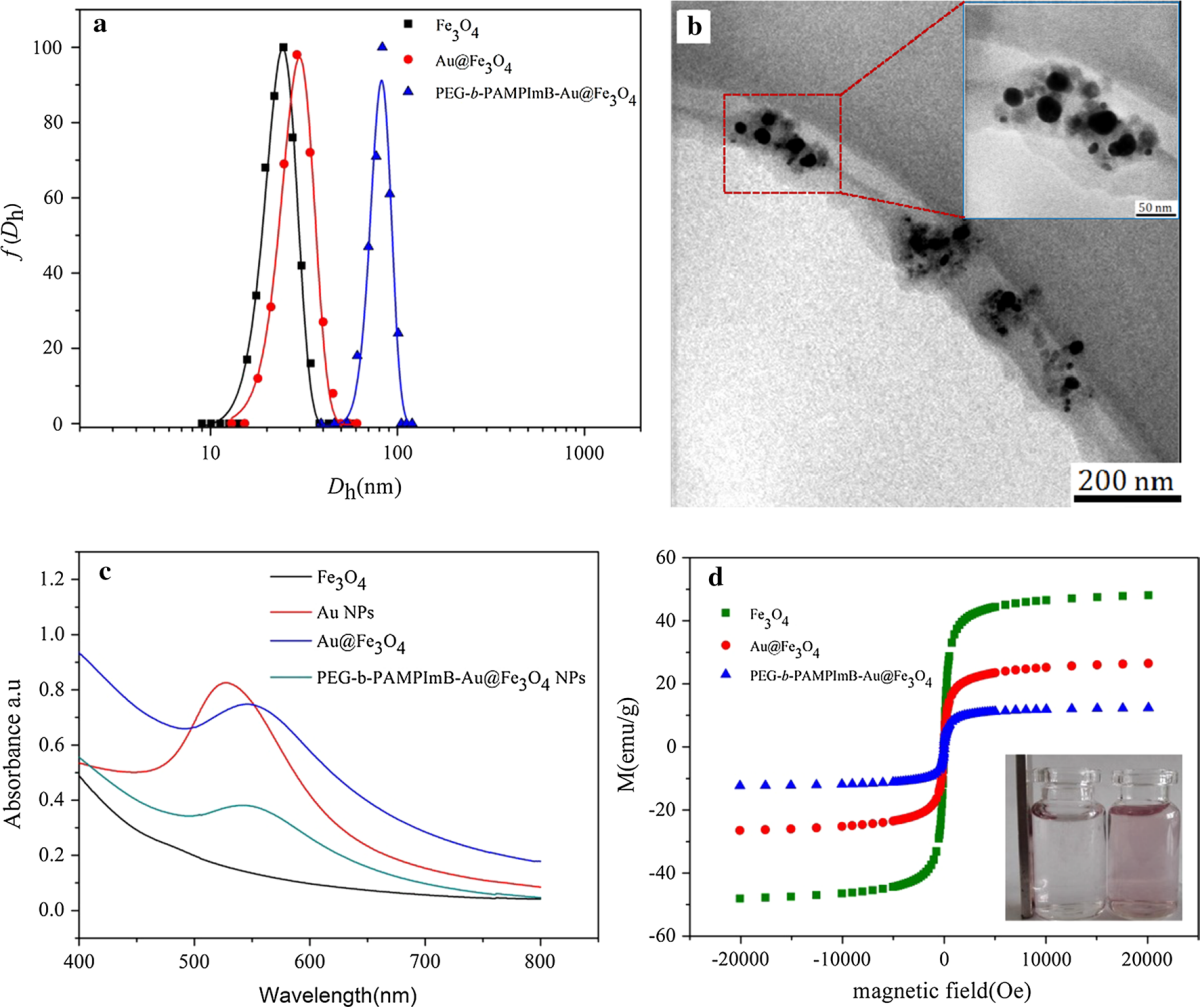
Hydrodynamic diameter distributions of Fe_3_O_4_ NPs, Fe_3_O_4_@Au NPs and PEG-*b*-PAMPImB-Fe_3_O_4_@Au NPs (**a**); TEM image of PEG-*b*-PAMPImB-Fe_3_O_4_@Au NPs (**b**); the inserted image is a magnification of the NPs in **b**; UV–vis spectra of Fe_3_O_4_ NPs, Au NPs, Fe_3_O_4_@Au NPs and PEG-*b*-PAMPImB-Fe_3_O_4_@Au NPs (**c**); magnetization measurements as a function of applied field for Fe_3_O_4_ NPs, Fe_3_O_4_@Au NPs and PEG-*b*-PAMPImB-Fe3O4@Au NPs (**d**). The inserted image in **d** is a photograph of a solution of PEG-b-PAMPImB-Fe_3_O_4_@Au NPs before (*right bottle*) and after (*left bottle*) applying a NdFeB magnet during 30 min

The hydrodynamic diameter and distribution of Fe_3_O_4_, Fe_3_O_4_@Au and PEG-*b*-PAMPImB-Fe_3_O_4_@Au NPs were first measured by dynamic light scattering (DLS). The average hydrodynamic diameter (*D*_h_) of the Fe_3_O_4_ NPs is approximately 21 nm and the size distribution range is 12–34 nm (Fig. 1a). The Fe_3_O_4_@Au NPs has an approximate *D*_h_ at 29 nm, which is larger than that of Fe_3_O_4_ NPs, thus confirming the formation of a thin Au shell. The mean thickness of the Au shell was estimated to be 4 nm. The larger *D*_h_ of PEG-*b*-PAMPImB-Fe_3_O_4_@Au NPs (83 nm) and their size distribution range (61–107 nm) are indicative of a successful polymer conjunction. PEG-*b*-PAMPImB-Fe_3_O_4_@Au NPs were further characterized by TEM (Fig. 1b). The clear covered polymer layers observed on the surface of Fe_3_O_4_@Au NPs (magnified image insert in Fig. 1b) further confirmed that PEG-b-PAMPImB attached onto Fe_3_O_4_@Au NPs, as these images are clearly different from TEM images of Fe_3_O_4_ (Additional file 1: Figure S1a) and Fe_3_O_4_@Au (Additional file 1: Figure S1b). UV–vis absorption of Fe_3_O_4_, Au, Fe_3_O_4_@Au and PEG-*b*-PAMPImB-Fe_3_O_4_@Au NPs revealed different spectrum properties (Fig. 1c). Indeed, no significant absorption peaks were detected in the visible light curve of Fe_3_O_4_ NPs. Pure Au NPs have clear surface plasmon resonance (SPR) absorption (525 nm). A similar SPR absorption (548 nm) was observed on the spectrum of Fe_3_O_4_@Au NPs, confirming that the Au shell formed successfully on the surface of Fe_3_O_4_ NPs [42]. Upon coating with PEG-*b*-PAMPImB, Fe_3_O_4_@Au NPs showed a minor blue shift in the SPR, from 548 to 542 nm, likely due to their enhanced dispersion in water. The superparamagnetic properties of Fe_3_O_4_, Fe_3_O_4_@Au and PEG-*b*-PAMPImB-Fe_3_O_4_@Au NPs were further assessed by Magnetization curves (M–H loop) measured at RT (Fig. 1d). The saturation magnetization value (Ms) for Fe_3_O_4_ NPs is 48.05 emu/g. After coating with the Au shell and PEG-*b*-PAMPImB, the Ms for Fe_3_O_4_@Au and PEG-*b*-PAMPImB-Fe_3_O_4_@Au NPs was lower, at 26.45 and 12.33 emu/g, respectively. This decrease in Ms has been attributed to the packaging of non-magnetic Au shell and PEG-*b*-PAMPImB on the periphery of Fe_3_O_4_ NPs [43]. The inserted photograph showed that PEG-*b*-PAMPImB-Fe_3_O_4_@Au NPs form a purple aqueous solution with homogeneous dispersion. This solution showed a typical macroscopic appearance of aggregation after positioning with a magnet for 30 min, demonstrating that PEG-*b*-PAMPImB-Fe_3_O_4_@Au NPs possess magnetic responsiveness.

### DNA loading capability of PEG-*b*-PAMPImB-Fe_3_O_4_@Au

**Fig. 2:**
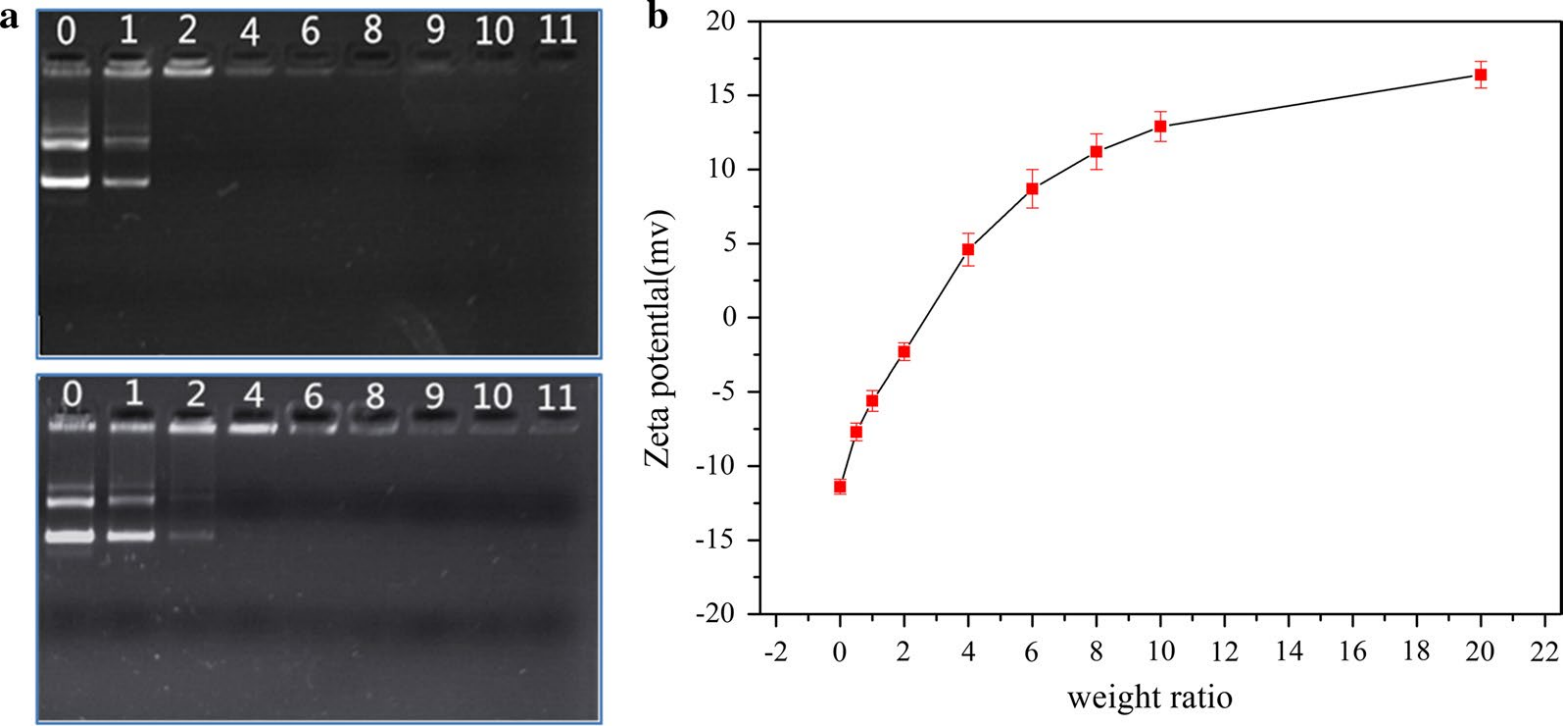
Gel migration assay for PEG-*b*-PAMPImB (*up*) and PEG-*b*-PAMPImB-Fe_3_O_4_@Au (*down*) complex with DNA at various weight ratios (**a**), where the number on *each lane* represents the ratio of PEG-*b*-PAMPImB and PEG-*b*-PAMPImB-Fe_3_O_4_@Au to DNA in complexes; zeta potentials of PEG-*b*-PAMPImB-Fe_3_O_4_@Au/DNA complexes in pure water at various weight ratios (**b**). Values represent mean (±SD [n = 3])

PEG-*b*-PAMPImB-Fe_3_O_4_@Au NPs are designed to load DNA via an electrostatic attraction between the positively charged PAMPImB and the negatively charged DNA phosphate groups. Agarose gel retardation assays were performed to assess the gene condensing capacity of the magnetic NPs and of the surface polymer. The migration of naked DNA, PEG-b-PAMPImB/DNA and PEG-b-PAMPImB-Fe_3_O_4_@Au NPs/DNA complexes at weight ratios ranged from 0/1 to 11/1 were shown in Fig. 2a. Both polymer and magnetic particles could condense pDNA efficiently at low weight ratios. The migration of DNA in agarose gels was significantly retarded and remained above the weight ratio (±) of two for PEG-*b*-PAMPImB, and above four for PEG-*b*-PAMPImB-Fe_3_O_4_@Au NPs. In agreement with these results, the zeta potentials (Fig. 2b) appear to increase with the weight ratio. At the weight ratio of five, the zeta potential of PEG-*b*-PAMPImB-Fe_3_O_4_@Au NPs with DNA shifts to positive. At higher ratios, the zeta potentials reach a maximum plateau, because the DNA negative charges are rapidly neutralized by the NPs’ excess positive charges.

### Size and colloidal stability of magnetic nanoplexes

Vector stability under physiological conditions has significant effects on gene expression in vitro and on further applications in vivo [44]. Our previous reports demonstrated that polyplexes [45] and nanoplexes [24] with a PEG shell, and which condense DNA via PAMPImB, are highly stable in physiological media. We assessed magnetic nanoplex stability in different media by DLS (Table 1). PEG-b-PAMPImB-Fe_3_O_4_@Au NPs were mixed with pGFP-C1 at weight ratios of 20/1, 10/1 or 5/1 in pure water, and then each mix was transferred into PBS buffer (20 mM, pH 7.4), 150 mM NaCl or 10% FBS solution. The DNA weight was fixed at 200 ng in every sample. Three magnetic nanoplexes show a small hydrodynamic size at 75, 87 and 101 nm, and narrow particle size distribution in pure water. The magnetic nanoplexes have the most similar size (±18 nm) to magnetic NPs without DNA (Fig. 1a), indicating that PEG-*b*-PAMPImB-Fe_3_O_4_@Au NPs can be used as template, based on core–shell structure, to monitor nanoplex size. The *D*_h_ and PDI of the nanoplexes, regardless of their weight-ratio, were nearly unchanged in PBS buffer (pH 7.4) and 150 mM NaCl solution, when compared to pure water. This result shows that the nanoplexes are stable in these physiological media. However, in 10% FBS the *D*_h_ of the magnetic nanoplexes increased slightly and the PDI broadened at every weight-ratio, which may be due to protein adsorption onto the nanoplexes’ surface. These data demonstrate that the periphery of magnetic nanoplexes covered by electrostatically neutral PEG provides high colloid stability for vectors in physiological conditions (i.e. presence of salt and serum).

**Table 1:** The hydrodynamic diameter and polydispersity index of PEG-b-PAMPImB/DNA-Fe_3_O_4_@Au NPs at different ratios and media

Weight ratio	Pure water		PBS (pH 7.4)		150 mM NaCl		10% FBS	
	*D*_h_ (nm)	PDI	*D*_h_ (nm)	PDI	*D*_h_ (nm)	PDI	*D*_h_ (nm)	PDI
20/1	75	0.15	73	0.20	72	0.16	89	0.25
10/1	87	0.17	84	0.19	81	0.19	116	0.24
5/1	101	0.21	98	0.18	96	0.19	122	0.27

### Cytotoxicity of magnetic nanoparticles and nanoplexes

**Fig. 3:**
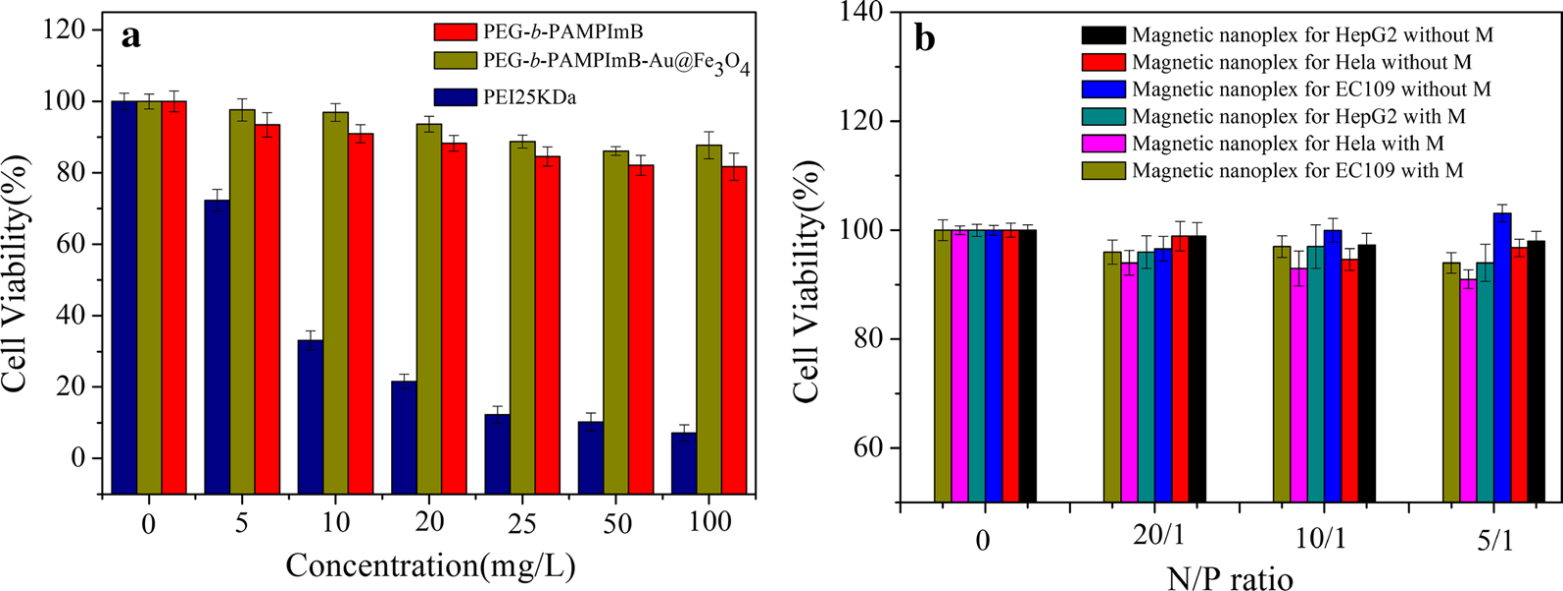
**a** Cytotoxicity of PEI25k, PEG-*b*-PAMPImB and PEG-*b*-PAMPImB-Fe_3_O_4_@Au in EC109 cells at various concentrations; **b** cytotoxicity of PEG-*b*-PAMPImB-Fe3O4@Au/DNA in HepG2, HeLa and EC109 cells at different weight ratios. Values represent mean (SD [n = 3])

Low cytotoxicity is a highly desired property for carriers in drug and gene delivery. We assessed cytotoxicity of PEI25k, PEG-*b*-PAMPImB and PEG-*b*-PAMPImB-Fe_3_O_4_@Au NPs in human esophageal cancer cells (EC109) with MTT assays, at various concentrations of vector (Fig. 3a). The blank test, which was considered as a positive control, showed 100% cell viability. PEG-*b*-PAMPImB and PEG-*b*-PAMPImB-Fe_3_O_4_@Au NPs exhibited significantly lower cytotoxicity than PEI25k, and PEG-*b*-PAMPImB-Fe_3_O_4_@Au NPs showed slightly higher biocompatibility than PEG-*b*-PAMPImB (Fig. 3a). The cytotoxicity of composite nanoparticles often depends on their size and surface properties [46]. PEG-*b*-PAMPImB is a low cytotoxic polymer because PEG and the cationic histamine-like segment have high biocompatibility, as shown in our previous report [45]. The formation of micelle-like core–shell structures upon attachment to Fe_3_O_4_@Au further decreases cytotoxicity, as the PEG outer corona shields the inner shell of the cationic histamine-like segment.

Finally, we assessed the cytotoxicity of the PEG-*b*-PAMPImB-Fe_3_O_4_@Au/DNA nanoplexes in HepG2, HeLa and EC109 cells in the presence or absence of a magnetic field (Fig. 3b). The magnetic nanoplexes showed significantly low cytotoxicity whether or not a magnetic field was applied to the cells, suggesting that exposure to a magnetic field does not affect normal cell proliferation.

### Magnetofection efficiency

**Fig. 4:**
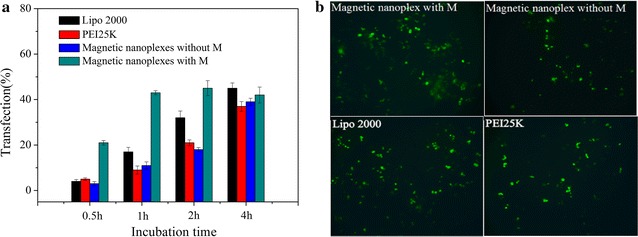
Transfection efficiency (% of cells transfected) of EC109 cells treated with pEGFP-C1 and magnetic nanoplexes with or without application of a magnetic field, PEI (25 kD) and Lipo2000 at various incubation times (**a**); fluorescence microscope images of the EC109 cells in **a** at 1 h of incubation (**b**). Values represent mean (SD [n = 3])

pEGFP-C1 was employed as a reporter gene for evaluating the transfection efficiency of the magnetic nanoplexes (at weight ratio of 5/1) in EC109 cells with or without application of a static magnetic field. Transfection of pEGFP-C1 with Lipofectamine2000 or PEI25K (N/P of 5/1) was used as positive control. Transfection efficiency was assessed by quantifying the GFP-expressing cells with flow cytometry. The cells were first incubated with the vectors in serum-free culture medium at 37 °C. This medium was then replaced with fresh medium containing 10% serum and the cells were incubated for 24 h at 37 °C. Figure 4a shows the percentage of cells transfected, at different incubation times. Notably, the magnetic nanoplex under a magnetic field shows significantly higher transfection efficiency at a shorter incubation time (0.5 and 1 h) than Lipofectamine2000, PEI25K and the magnetic nanoplex without application of a magnetic field. The magnetic transfection efficiency reached a maximum of about 43% after 1 h of incubation, which is fivefold, threefold, and fourfold higher than the transfection efficiency of the nanoplexes without a magnetic field, Lipofectamine2000, and PEI25K, respectively. Moreover, the transfection efficiency of pEGFP-C1 with Lipofectamine2000, PEI25K and the magnetic nanoplex without a magnetic field increased with the incubation time, consistent with standard transfections [47]. Direct observation of the transfected cells with an inverted fluorescence microscope revealed that magnetofection at 1 h of incubation has the highest transfection activity when compared to transfections in the other conditions (Fig. 4b). These results suggest that the rapid accumulation of DNA-carrying magnetic vectors around cells increases transfection efficiency.

### Cell uptake and magnetic separation

**Fig. 5:**
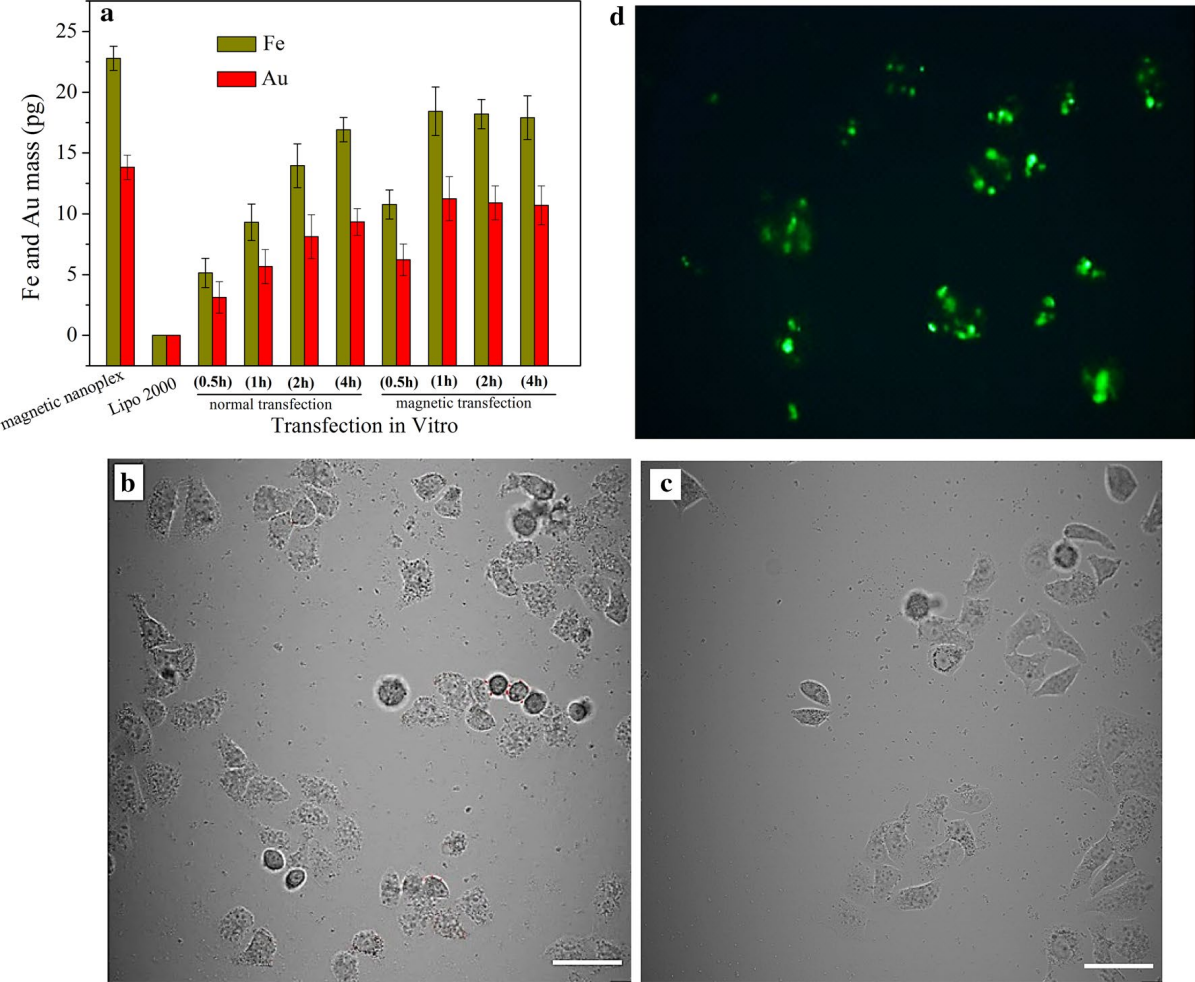
The Fe and Au content in transfected cells at various incubation times (**a**); confocal microscopic images of EC109 cells transfected with magnetic nanoplex with (**b**) and without the application of a magnetic filed (**c**) at 1 h incubation time (*scale bar* is 50 μm); fluorescence microscope image of EC109 transfected cells after magnetic collection and growth overnight (**d**). Values represent mean (SD [n = 3])

The mean mass of Fe and Au in total EC109 cells was measured by inductive coupled plasma-mass spectrometry (ICP-MS) at different incubation times (Fig. 5a). Before transfection, the magnetic nanoplexes contained 22.8 pg Fe and 13.8 pg Au. At 0.5–2 h of incubation time, the cells transfected using magnet-assisted transfection had a higher mass of Fe and Au than those transfected using a standard transfection method. The content of Fe and Au in the magnetotransfected cells peaked at 1 h and then remained unchanged, suggesting that internalization in the presence of a magnetic field was completed at this time point. Consistent with the continuous increase in uptake rate over time that is typical of standard transfections, the mass of Fe and Au in cells transfected with Lipofectamine2000, PEI25K or magnetic nanoplexes without a magnetic field at 4 h was comparable to the internalization rates of magnetic transfections at 1 h. Confocal laser scanning microscopy (CLSM) revealed that there is significantly more internalization of magnetic nanoplexes in transfections with the application of an external magnetic field (Fig. 5b) than without (Fig. 5c), in agreement with the ICP-MS results above. The accumulation of magnetic NPs in transfected cells confers them magnetic properties, thereby allowing for selective cell separation by application of a magnetic field [48]. After harvesting with an external magnetic field, the transfected cells were incubated overnight and then observed on an inverted fluorescence microscope. Compared with the result in Fig. 4b, a clear increase in fluorescence intensity could be detected due to the magnetic agglomeration of transfected cells (Fig. 5d).

Magnetic gene delivery systems have attracted wide interesting because of their potential to achieve selective and efficient delivery of therapeutic genes to the target site/cells. Commonly, most formulations were fabricated with a magnetic core for magnetic target function and external coating cationic polymer for loading therapeutic gene. For example, Shi et al. conjugated plasmids on the surface of PEI modified Au–Fe_3_O_4_ dumbbell nanoparticles and obtained high efficiency in transfecting adherent mammalian cells under the magnetic attraction [49]. Zamanian’ group prepared chitosan coated Fe_3_O_4_ nanoparticles and demonstrated the particles can enhance magnetofection efficiency due to the advantages posed by its magnetic properties and DNA-binding ability [33]. Those formulations have been widely demonstrated a high transfection efficiency in vitro by application of external magnetic field. However, the undesired aggregation was also brought due to random interactions between the vector’s positive surface and the negative charges of biomacromolecules or components in physiological media [19], as these aggregated vectors would be unacceptable for medical applications in vivo.

In order to improve the applicability of magnetic delivery system, we developed Fe_3_O_4_@Au heterogeneous NPs capped neutral-cationic block copolymer as DNA vectors for magnetofection assays. In contrast to reported magnetic formulations, our vector shows clear benefits. First, we used Au-coated Fe_3_O_4_ heterogeneous NPs (Fe_3_O_4_@Au) as magnetic core instead of single Fe_3_O_4_ nanoparticles. The Au shell of Fe_3_O_4_@Au would increase Fe_3_O_4_ core stability and biocompatibility, while provide a platform for easily achieving covalent modifications via Au–S chemistry [50]. Second, we designed a neutral-cationic block copolymer as external coating polymer rather than only cationic polymer monolayer. The outer neutral PEG can provide high colloidal stability of vector in physiological conditions [45]. Moreover, the magnetic agglomeration of transfected cells has been proven to be feasible.

## Conclusion

We have developed Fe_3_O_4_@Au heterogeneous NPs capped with neutral-cationic block copolymer via Au–S covalent bonds, and assessed their feasibility as DNA vectors in magnetofection assays. This vector has a four-layer microstructure comprising a Fe_3_O_4_ core, an Au shell, an inner cationic polymer and an outer neutral PEG. These different layers provide well-defined functions for magnetic response, polymer conjunction, DNA loading and colloidal stability. We show that the magnetic nanoplexes have high stability in physiological conditions and are rapidly internalized in magnet-assisted transfections, thereby allowing for efficient separation of transfected cells. Thus, precisely engineered architectures based on neutral-cationic block copolymer-conjugated heterogeneous NPs provide a valuable strategy for improving the efficacy and applicability of synthesized vectors.

## Methods

### Materials

Borane-tert-butylamine complex (BTBA), hydrogen tetrachloroaurate (HAuCl_4_·3H_2_O) and branched poly(ethylenimine) (PEI25k) were purchased from Sigma-Aldrich and used as received. Ferrous chloride tetrahydrate (FeCl_2_·4H_2_O, >99% purity) and ferric chloride hexahydrate (FeCl_3_·6H_2_O, >99% purity) were bought from Shanghai Chemical Reagent Co., Ltd. (Shanghai, China). PEG_112_-*b*-PAMPImB_96_ was synthesized by RAFT polymerization by using PEG-CTA (Mw 5000) as macromolecular chain transfer agent and AMPImB as monomer. The detailed synthesis and characterization of this polymer was previously described [45]. Dulbecco’s modified Eagle’s medium (DMEM), penicillin–streptomycin, trypsin, fetal bovine serum (FBS), 3-[4,5-dimethylthiazol-2-yl]-2,5-diphenyltetrazolium bromide (MTT), and Dubelcco’s phosphate buffered saline (DPBS) were purchased from Thermo Fisher Scientific. The reporter plasmid, enhanced green fluorescent protein gene (pEGFP-C1), was amplified in *E. coli* and purified by E.Z.N.A.^®^ Endo-free plasmid DNA maxi kit (Omega). Purified pEGFP-C1 was stored at −20 °C and thawed at RT for the transfection assays.

### Preparation of Fe_3_O_4_@Au nanoparticles

Magnetic nanoparticles of Fe_3_O_4_ were prepared with a chemical coprecipitation method according to a previously reported procedure [38]. Briefly, FeCl_2_·4H_2_O (0.398 g, 2.5 mmol) and FeCl_3_·6H_2_O (1.352 g, 5 mmol) were dissolved in 100 mL of Milli-Q water containing 20 µL of concentrated HCl. The solution was heated to 80 °C under a nitrogen atmosphere and then 150 mL of sodium hydroxide (1 M) was added dropwise with vigorous stirring. After stirring for an hour, the magnetic nanoparticles were harvested by using an NdFeB magnet, and washed with Milli-Q water 3–4 times until the supernatant liquor reached neutrality. Finally, the resulting Fe_3_O_4_ NPs were dried under vacuum at 60 °C for further use.

Au-coated Fe_3_O_4_ NPs (Fe_3_O_4_@Au) were prepared by directly reducing HAuCl_4_ on the surface of APTES-functionalized Fe_3_O_4_ NPs. 0.2 g of Fe_3_O_4_ NPs were ultrasonically dispersed in 50 mL anhydrous ethanol, and then 0.1 mL of APTES as added at room temperature (RT). The mixture was vigorously stirred for 24 h and then acidized by adding 0.05 mL of a concentrated HNO_3_ solution. APTES functionalization of Fe_3_O_4_ NPs was carried out in three cycles of separation and wash by using an NdFeB magnet and ethanol, respectively. The product was mixed with a 1% HAuCl_4_ ethanol solution followed by dropwise addition of BTBA (0.05%, w/v). The color of the solution changed from brown to reddish-brown and then purple as the Au content was increased. The Fe_3_O_4_@Au NPs were purified by magnet separation and washed with 0.1 M HCl to remove the free Au and Fe_3_O_4_ NPs. Finally, the particles were re-dispersed in ethanol for further use.

### Preparation of PEG-*b*-PAMPImB-Fe_3_O_4_@Au nanoparticles

PEG-*b*-PAMPImB-modified Fe_3_O_4_@Au (PEG-*b*-PAMPImB-Fe_3_O_4_@Au) NPs were prepared via formation of Au–S covalent bonds between the terminated group of polymer and an Au shell layer of Fe_3_O_4_@Au. We used a classic procedure as follows: PEG_112_-*b*-PAMPImB_96_ (0.1 g) was added to a 100 mL round-bottom flask containing 10 mL of an Fe_3_O_4_@Au ethanol suspension; 1 mL of 0.1 M aqueous hydrazine solution was then added with vigorous stirring to reduce its dithioester-terminated group with thiol. After 3 days of equilibrium at RT, the formed PEG-*b*-PAMPImB-Fe_3_O_4_@Au NPs were collected with a magnet and washed with water to remove unbound polymer. After vacuum drying, 0.1 g of PEG-*b*-PAMPImB-Fe_3_O_4_@Au was dissolved in 100 mL of Milli-Q water for further use.

### Preparation of PEG-*b*-PAMPImB-Fe_3_O_4_@Au/DNA nanoplexes

PEG-*b*-PAMPImB-Fe_3_O_4_@Au NPs bound to DNA at various weight ratios were prepared by adding different volumes of PEG-*b*-PAMPImB-Fe_3_O_4_@Au (1 mg/mL) and 36 µL of DNA plasmid (200 ng/µL) into an aqueous solution. The nanoplexes (PEG-*b*-PAMPImB-Fe_3_O_4_@Au/DNA) were gently vortexed and then incubated for 30 min at RT to ensure stable formation of nanoplexes. The nanoplexes were then subjected to a centrifuging–redispersing process to remove free unbound DNA.

### Cell culture

EC109 cells were cultured in DMEM medium supplemented with 10% (v/v) heat-inactivated FBS and 1% penicillin–streptomycin at 37 °C in a humidified atmosphere containing 5% CO_2_.

### Agarose gel retardation assay

The nanoplexes’ condensation capability was assessed by agarose gel electrophoresis. NP/DNA nanoplexes with serial weight ratios ranging from 0/1 to 10/1 were prepared according to the conditions described above. After 30 min of incubation at RT, the nanoplex solutions were analyzed by 1% agarose gel electrophoresis (100 V, 30 min) in TAE buffer. The DNA bands were visualized with UV light and analyzed with Cam2com software.

### Cytotoxicity assay

EC109 cells were seeded into 96-well plates at 5000 cells/well and cultured 24 h in 200 μL of DMEM containing 10% FBS. A range of concentrations of PEG-*b*-PAMPImB, PEG-*b*-PAMPImB-Fe_3_O_4_@Au and PEI25k were prepared in PBS solution (pH 7.4). To estimate the influence of an external magnetic field on cell viability, PEG-*b*-PAMPImB-Fe_3_O_4_@Au/DNA nanoplexes with DNA weight ratios of 20/1, 10/1 and 5/1 were prepared and added into the wells. A magnetic sheet was placed under well plate to apply the magnetic field. 20 μL of each solution was added to the corresponding well, followed by 24 h of incubation. Then, the medium was replaced with 200 μL of fresh medium. MTT (20 μL, 5 mg/mL in PBS) stock solution was then added to each well. After 4 h, unreacted dye was carefully removed, and the formazan crystals were dissolved in DMSO (200 μL/well). The plates were incubated for another 10 min before measuring the absorbance at 570 nm with an ELISA microplate reader (Bio-Rad). Cell viability (%) was calculated as previously described.

### In vitro transfection

To assess the transfection activity of the nanoplexes, EC109 cells were seeded in 24-well plates with an initial density of 5 × 10^4^ cells/well in 1 mL DMEM containing 10% FBS and then incubated at 37 °C for 24 h in 5% CO_2_ (to reach 70% confluence at the time of transfection). The magnetic nanoplexes, PEI25k/DNA and Lipofectamine2000/DNA were added to wells containing serum-free culture medium at 37 °C. After incubation, the medium was replaced with fresh medium containing 10% serum and transfected for 48 h at 37 °C. Magnetofection was performed by placing a magnetic sheet under the plates. The cells were monitored for expression of green fluorescence protein (GFP) with a fluorescence microscope. For observation with a confocal laser scanning microscope (LSM 780, Zeiss), the cells were washed with PBS three times, fixed with 4% paraformaldehyde for 30 min. Transfection efficiency was determined by flow cytometry to quantify the percentage of GFP-expressing cells. After transfection for 48 h at 37 °C, the harvested cells were washed with PBS, detached with 0.25% trypsin and then resuspended in 500 μL PBS (pH 7.4) for flow cytometry (FC500, Beckman Coulter).

To quantify the intracellular uptake of magnetic NPs, ICP-MS was performed to measure the concentration of Fe and Au in total cells. After incubation, the medium was removed and the cells were washed with PBS three times, and then treated with trypsin solution (containing 0.25% EDTA). The cell pellets were sorted into a 20 mL silicon glass vial and completely digested with 500 µL of Aqua regia. The digested solution was diluted to 5 mL with 1% Aqua regia and filtered with 0.22 μm filters (Millipore, USA). For estimating percentage of uptake, the same dosage of magnetic nanoplexes was directly digested by Aqua regia and diluted to the same volume for measuring Fe and Au concentration with ICP-MS (7500A, Agilent, USA).

### Magnetic collection of transfected cells

EC109 cells were transfected at weight ratio 5/1, as described above. After transfection for 48 h at 37 °C, the harvested cells were washed with PBS, detached with 0.25% trypsin and then resuspended in 500 μL PBS (pH 7.4). Magnetic collection of transfected cell was performed by placing a magnetic sheet under the plates during the free sedimentation of cells. After 15 min, the medium was carefully removed and the cells were washed with PBS three times. Finally, the transfected cells were further incubated overnight and observed directly with a fluorescence microscope.

### Characterization

Dynamic laser scattering (DLS) measurements were performed using a laser light scattering spectrometer (BI-200SM) equipped with a digital correlator (BI-9000AT) at 532 nm at RT. Transmission electron microscopy (TEM) measurements were conducted using a JEM-2100 electron microscope at an accelerating voltage of 200 kV; a small drop of solution was deposited onto a carbon-coated copper EM grid and dried at RT under atmospheric pressure. The UV–vis spectra were recorded on a Cary 50 Bio UV–Visible Spectrophotometer (Varian, USA) equipped with two silicon diode detectors and a xenon flash lamp. Zeta-potentials were measured using a temperature-controlled Zetasizer 2000 (Malvern Instruments. Ltd. UK).

## Additional file

**Additional file 1: Figure S1.** TEM images of Fe_3_O_4_ NPs (a) and Fe_3_O_4_@Au NPs (b).

## Abbreviations

Fe_3_O_4_@Au: Au coating Fe_3_O_4_ NPs
PEG-*b*-PAMPImB-Fe_3_O_4_@Au: polyglycol-*b*-poly1-(3-aminopropyl)-3-(2-methacryloyloxy propylimidazolium bromine)
EC109: human esophageal cancer cell line
Au NPs: gold nanoparticles
P(HPMA-*b*-DMAPMA): poly(*N*-2-hydroxypropyl methacrylamide-*block*-*N*-[3-(dimethylamino)propyl] methacrylamide)
Fe_3_O_4_ NPs: iron oxide magnetic nanoparticles
RAFT: reversible addition–fragmentation chain transfer
DLS: dynamic light scattering
*D*_h_: average hydrodynamic diameter
TEM: transmission electron microscopy
SPR: surface plasmon resonance
Ms: saturation magnetization value
ICP-MS: investigated by inductively coupled plasma mass spectrometry
CLSM: confocal laser scanning microscopy
BTBA: borane-tert-butylamine complex
PEI25k: branched poly(ethylenimine)
DMEM: Dulbecco’s modified Eagle’s medium
FBS: fetal bovine serum
MTT: 3-[4,5-dimethylthiazol-2-yl]-2,5-diphenyltetrazolium bromide
DPBS: Dubelcco’s phosphate buffered saline
pEGFP-C1: plasmid enhanced green fluorescent protein
RT: room temperature
