# Supplementary figures and images for: Block copolymer conjugated Au-coated Fe3O4 nanoparticles as vectors for enhancing colloidal stability and cellular uptake
Source: J Nanobiotechnology. 2017 Jul 25;15:56. doi: 10.1186/s12951-017-0290-5 (PMC5526242; doi:10.1186/s12951-017-0290-5)

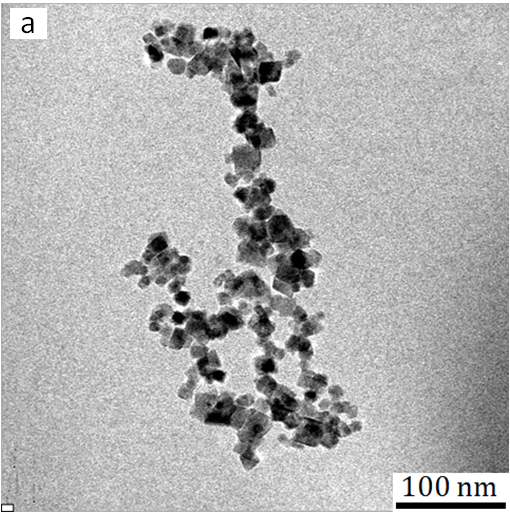

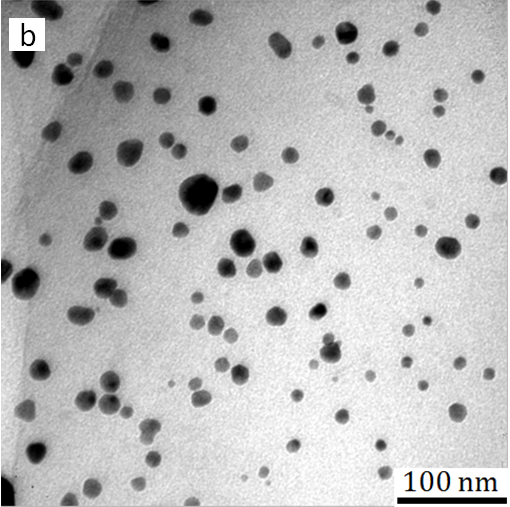


Figure S1. TEM image of Fe3O4 NPs(a) and Fe3O4@Au NPs (b).

Supplement: Supplementary file 1 — Additional file 1: Figure S1. TEM images of Fe3O4 NPs (a) and Fe3O4@Au NPs (b). [file 12951_2017_290_MOESM1_ESM.doc]
